# A multi-institutional machine learning algorithm for prognosticating facial nerve injury following microsurgical resection of vestibular schwannoma

**DOI:** 10.1038/s41598-024-63161-1

**Published:** 2024-06-05

**Authors:** Sabrina M. Heman-Ackah, Rachel Blue, Alexandra E. Quimby, Hussein Abdallah, Elizabeth M. Sweeney, Daksh Chauhan, Tiffany Hwa, Jason Brant, Michael J. Ruckenstein, Douglas C. Bigelow, Christina Jackson, Georgios Zenonos, Paul Gardner, Selena E. Briggs, Yale Cohen, John Y. K. Lee

**Affiliations:** 1 Department of Neurosurgery, Perelman Center for Advanced Medicine, University of Pennsylvania, 3400 Civic Center Boulevard, 15th Floor, Philadelphia, PA 19104 USA; 2 Department of Bioengineering, University of Pennsylvania, Philadelphia, PA USA; 3 Department of Otorhinolaryngology, University of Pennsylvania, Philadelphia, PA USA; 4 Department of Otolaryngology and Communication Sciences, SUNY Upstate Medical University Hospital, Syracuse, NY USA; 5 School of Medicine, University of Pittsburgh, Pittsburgh, PA USA; 6 Department of Biostatistics, Epidemiology and Informatics, University of Pennsylvania, Philadelphia, PA USA; 7 University of Pennsylvania, Perelman School of Medicine, Philadelphia, PA USA; 8 Corporal Michael J. Crescenz VAMC, Philadelphia, PA USA; 9 Center for Cranial Base Surgery, University of Pittsburgh, Pittsburgh, PA USA; 10 Department of Otolaryngology, MedStar Washington Hospital Center, Washington, DC USA; 11 Department of Otolaryngology, Georgetown University, Washington, DC USA

**Keywords:** Outcomes research, Risk factors, Machine learning

## Abstract

Vestibular schwannomas (VS) are the most common tumor of the skull base with available treatment options that carry a risk of iatrogenic injury to the facial nerve, which can significantly impact patients’ quality of life. As facial nerve outcomes remain challenging to prognosticate, we endeavored to utilize machine learning to decipher predictive factors relevant to facial nerve outcomes following microsurgical resection of VS. A database of patient-, tumor- and surgery-specific features was constructed via retrospective chart review of 242 consecutive patients who underwent microsurgical resection of VS over a 7-year study period. This database was then used to train non-linear supervised machine learning classifiers to predict facial nerve preservation, defined as House-Brackmann (HB) I vs. facial nerve injury, defined as HB II–VI, as determined at 6-month outpatient follow-up. A random forest algorithm demonstrated 90.5% accuracy, 90% sensitivity and 90% specificity in facial nerve injury prognostication. A random variable (rv) was generated by randomly sampling a Gaussian distribution and used as a benchmark to compare the predictiveness of other features. This analysis revealed age, body mass index (BMI), case length and the tumor dimension representing tumor growth towards the brainstem as prognosticators of facial nerve injury. When validated via prospective assessment of facial nerve injury risk, this model demonstrated 84% accuracy. Here, we describe the development of a machine learning algorithm to predict the likelihood of facial nerve injury following microsurgical resection of VS. In addition to serving as a clinically applicable tool, this highlights the potential of machine learning to reveal non-linear relationships between variables which may have clinical value in prognostication of outcomes for high-risk surgical procedures.

## Introduction

Vestibular schwannomas (VS; formerly acoustic neuroma) are the most common tumor of the skull base with nearly 2500 new cases diagnosed in the US each year^1^ and accounting for 8% of all intracranial tumors^2^. VS continue to present a clinical conundrum in that their benign pathology affords a slow growth pattern with low likelihood of metastasis; however, local compression of cranial nerve VIII (the vestibulocochlear nerve) and the brainstem can result in hearing loss, dizziness, vertigo and in the worst cases, sudden death^3^. Furthermore, microsurgical resection carries an inherent risk of iatrogenic injury to each of these structures with similar clinical consequences. Microsurgery carries an additional risk of injury to the nearby cranial nerve VII (facial nerve) which may result in significant morbidity and impairment in quality of life. As such, the likelihood of damage to each of these structures from treatment must be weighed against the likelihood of developing complications from the natural history of tumor progression.

Historically, treatment decisions have been based on tumor size and growth patterns over time. Treatment is often considered when serial growth is observed on interval imaging, or patients develop neurological symptoms that correlate with tumor compression. Microsurgery remains the mainstay of treatment for large VS^4^. For lesions > 2.5 cm, vestibulocochlear nerve compression beyond salvageability is often encountered and patients are preemptively counseled that hearing preservation is unlikely^5^. However, facial nerve preservation remains an important goal of surgery. While larger tumors are generally associated with more difficult facial nerve dissection, few other factors that portend a higher risk of facial nerve dysfunction have been identified, and thus prognostication at the individual patient level remains relatively poor^6^. Even in situations where anatomic preservation of the nerve is achieved, stretching or other trauma from difficult dissection can lead to post-operative facial weakness. While machine learning algorithms have recently been developed in the domain of VS to aid in decisions regarding timing of treatment^7^ and likelihood of hearing preservation^8^, no study has yet applied this technology to discern the likelihood of facial nerve dysfunction, nor to develop a deeper understanding of the clinically relevant factors which may contribute to poorer facial nerve outcomes. We leveraged emerging machine learning approaches combined with the VS experience at two high-volume VS centers to develop an algorithm for prediction of facial nerve dysfunction in patients undergoing microsurgical resection of VS, based on patient, surgery, and tumor characteristics.

## Methods

### Database collection

This study was reviewed by the University of Pennsylvania Institutional Review Board, who determined that it met criteria for exemption from full ethical approval and subject informed consent. All methods were carried out in accordance with relevant guidelines and regulations. Data from the University of Pittsburgh were shared in accordance with executed Data Usage Agreements between the University of Pittsburgh and the University of Pennsylvania. We conducted a retrospective chart review of patients who had undergone retrosigmoid or translabyrinthine craniotomies for VS over a seven-year study period from 2014 to 2021 at three hospitals: the Hospital of the University of Pennsylvania, Pennsylvania Hospital, and the University of Pittsburgh Medical Center Presbyterian Hospital. We excluded patients who underwent retrosigmoid or translabyrinthine craniotomies for other pathologies, including lower cranial nerve schwannomas, meningiomas, chordomas, epidermoid cysts and brain metastasis. Two patients expired prior to the 6-month follow-up period and are thus not represented in this analysis. Data reviewed included patient demographic information, surgical reports, and pre- and post-operative magnetic resonance images.

### Statistical methods

The primary outcome assessed was facial nerve function at 6-month follow-up. Facial nerve function was assessed on the basis of physician ratings of facial function, measured using the House-Brackman (HB) scale at 6-month post-operative follow-up visits. This was represented as a binary outcome variable, post-operative preserved facial nerve function (HB grade I) vs. post-operative facial nerve dysfunction (HB grades II–VI). Independent variables included patient-, tumor- and surgery-related characteristics, as described below.

Measurements of tumor dimensions were made relative to the porus acusticus and posterior petrous bone (Supplementary Fig. 1)^9^. These dimensions were selected due to their relationships to surgical corridors and in keeping with the goal of reproducibility in replicative efforts. Such measurements have also been shown to correlate well with volumetric analyses^10^. Measurements were made by two raters, and agreement was assessed by intraclass correlation coefficient (ICC) accounting for 2-way random effects^11^.

Normality of continuous variables was assessed using D’Agostino-Pearson’s test^12^, finding that measurement C and case length were normally distributed, and thus were compared between HB I and HB II–VI groups with independent samples t-test. In contrast, age, BMI, measurements A, B, and D were not normally distributed and thus statistical significance of comparisons between facial nerve outcome groups was assessed using a Mann–Whitney U test. Categorical variables (sex, laterality, tumor size represented as a binary measurement of ≥ 2.5 cm vs. < 2.5 cm greatest tumor dimension, and presence/absence of residual tumor) were evaluated for associations to the outcome using Chi-squared tests. All statistical tests were evaluated at a significance level of alpha = 0.05.

### Machine learning classifier selection and training

Studies of machine learning proceed through certain regimented stages known as the machine learning lifecycle^13^. Although variations may exist based on the specific study and goals, in general, the lifecycle starts with data collection and pre-processing before proceeding through gathering of baseline descriptive statistical analysis (described above), classifier selection, model training, hyperparameter tuning, model testing and ultimately deployment with the subsequent collection of additional training examples for validation during deployment re-starting the cycle at data collection (Supplementary Fig. 2).

To guide classifier selection, the data were first visualized by class distribution on each feature axis using a pairplot (Supplementary Fig. 3). This demonstrated two important characteristics of the data: the class imbalance was likely significant enough to influence classifier performance and the data were not linearly separable on any two-dimensional feature axis plane. Given the relatively small size of the dataset, we applied the synthetic minority oversampling technique (SMOTE)^14^ to overcome class imbalance: this provided new training examples that would be useful in classifier training while equalizing the class distribution. Model training then proceeded with selection of a classifier that was suitable for the classification task while taking into account the restraints of the data. Because the data were not linearly separable, we selected non-linear classifiers, including the random forest, radial basis function (RBF) kernel support vector machine (SVM), and artificial neural network^13^ (Supplementary Fig. 2). Among these, the random forest classifier was selected for further development due to its superior accuracy in performing the classification task on the training data. The data were split for model training (90%) and subsequent testing (10%). While model tuning was attempted via hyperparameter optimization, the initial random forest model with hyperparameters based on the authors’ prior experience with similar classification tasks and patient datasets demonstrated the highest accuracy.

The validation dataset (n = 32 patients) consisted entirely of patients who underwent surgery at the University of Pennsylvania in the final year of the study, as this group had facial nerve outcomes assessed after initial algorithm development and thus were not included in the initial training and testing data sets. The same patient, tumor and surgery characteristics were collected for the 32 validation patients and the random forest algorithm was utilized to make predictions about which patients would have complete facial nerve preservation vs. those who would have any facial nerve dysfunction. Predicted outcomes were recorded and compared to actual 6-month facial nerve outcomes for this group of patients.

## Results

Two-hundred and forty-two consecutive patients were identified who underwent microsurgical resection of VS over the specified time period. Of these, 206 (85%) had preserved facial nerve function (HB I), and 36 (15%) had any facial nerve dysfunction (HB II–VI). Summary statistics and tests of association for underlying differences in patient-, tumor-, and surgery-specific characteristics between outcome groups are shown in Table 1. Among the factors evaluated, none demonstrated a statistically significant difference between the HB I and HB II–VI groups when evaluated on the basis of linear comparisons of measures of centrality (i.e., means and medians). The ICC for tumor measurements was between 90 and 99% for all measurements (Supplementary Fig. 1, Supplementary Table 1).

**Table 1:** Summary statistics.

	HB I	HB II–VI	p-value
Total patients	206 (85%)	36 (15%)	
Median age (IQR)	52.75 (20.67)	58.12 (14.44)	0.18
Median BMI (IQR)	28.50 (8.77)	30.43 (6.90)	0.09
Sex			0.30
Male (%)	92 (45%)	20 (55%)	
Female (%)	114 (55%)	16 (44%)	
Laterality			0.20
R (%)	93 (45%)	21 (58%)	
L (%)	113 (55%)	15 (42%)	
Tumor size			0.99
≥ 2.5 cm	77 (37%)	14 (39%)	
< 2.5 cm	129 (63%)	22 (61%)	
Measurement A median (IQR)	20.70 (16.13)	21.70 (14.38)	0.23
Measurement B median (IQR)	9.25 (7.88	10.55 (7.55)	0.24
Measurement C mean (SD)	6.50 (4.19)	6.48 (4.15)	0.20
Measurement D median (IQR)	8.95 (4.78(	10.05 (5.90)	0.61
Presence of residual			0.38
Yes (%)	63 (31%)	10 (28%)	
No (%)	143 (69%)	26 (72%)	
Approach			1.00
TL (%)	63 (31%)	11 (31%)	
RS (%)	143 (69%)	25 (69%)	
Average case length (SD)	477.11 (171.88)	522.5 (127.36)	0.07

**Figure 1:**
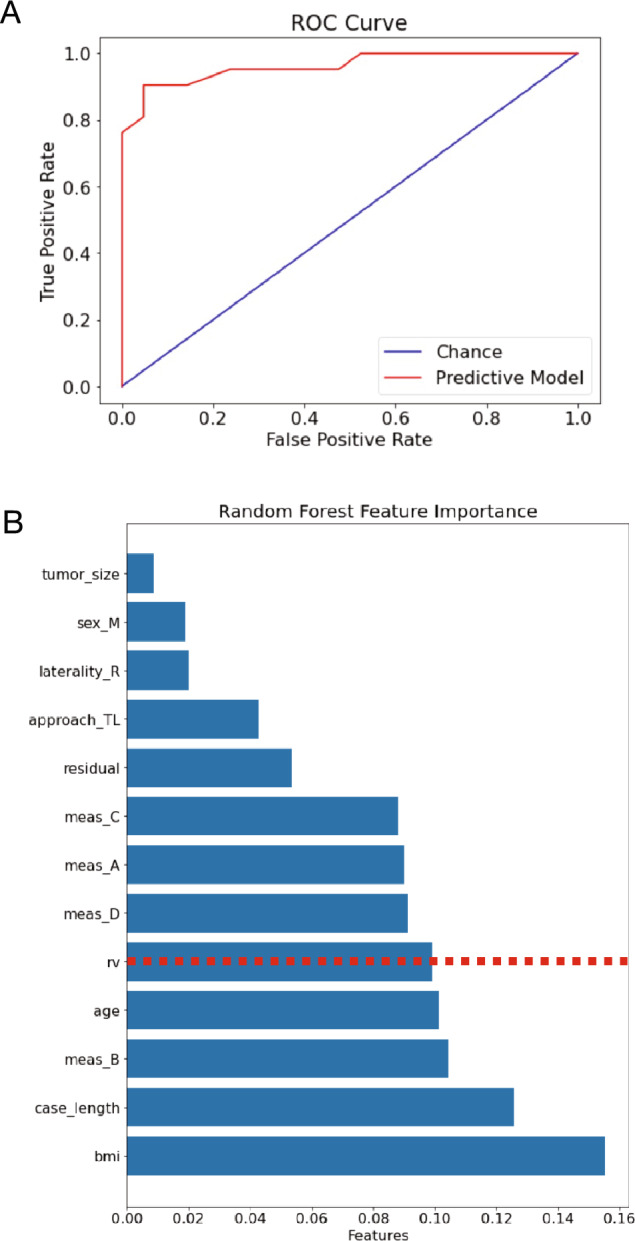
Random forest model evaluation. (**A**) A receiver-operating characteristic (ROC) curve of model performance on the test dataset was generated, demonstrating good performance of the random forest model. (**B**) Random forest feature importances were computed and graphed. Interestingly, BMI, case length, age, and the tumor dimension representing growth towards the brainstem (measurement B) were found to be most important for prediction of facial nerve outcomes.

When visualized in two dimensions, our data were not found to have linearly separable hyperplanes along any of the acquired feature axes (Supplementary Fig. 3). As such, non-linear supervised machine learning classifiers were tested as described in Methods (see also Supplementary Fig. 2). The random forest classifier performed well with an accuracy of 90.5%^15^. Given the goal of applying the classifier as a clinical tool, sensitivity and specificity were assessed on the test data, and were found to be 90% and 90%, respectively. The receiver-operating characteristic (ROC) curve is shown in Fig. 1A. A random sampling from a Gaussian distribution was generated as a random variable and used as a baseline to further evaluate which features were relevant in the random forest predictions: the resulting feature importances were computed and plotted (Fig. 1B). Relative to this baseline, the random forest classifier indicated a relatively greater importance of BMI, case length, age, and measurement B, representing the extent of brainstem compression, in facial nerve function prognostication. When tested on the validation data set, the model demonstrated 84% accuracy in predicting facial nerve function at 6 months post-operatively.

## Discussion

Facial nerve injury is a morbid complication of treatment for VS, with downstream effects ranging from social stigmata, patient depression and reduced quality of life^16,17^, to corneal abrasions and ulcers from incomplete eye closure and loss of corneal sensation^18^. Other than tumor size, relatively little is understood about factors that may influence facial nerve outcomes in microsurgery for VS. The clinical impact of facial nerve injury and importance of facial nerve preservation is highlighted by the extensive literature exploring predictors of facial nerve injury^19–23^. We leveraged our multi-institutional experience at two centers with high volumes of VS patients and applied machine learning techniques to identify novel predictors of facial nerve injury in patients treated with microsurgery.

Machine learning technologies have recently undergone a resurgence alongside the development of computational tools for handling and storing the large amounts of data required for their meaningful and broad scale utilization^13,24^. The recognition that such tools can be used to glean novel trends from data that are not readily apparent from common descriptive statistical approaches makes their application within the clinical domain a valuable and ongoing endeavor^25^. Such a phenomenon can be seen in the present study where tests of association, comparing measures of centrality between outcome groups, did not identify any factors which significantly differed between patients with and without preserved facial function. In contrast, random forest feature importance analysis discerned four features—BMI, case length, age and the tumor dimension representing growth towards the brainstem (measurement B)—as being relevant in predicting 6-month facial nerve status. While further studies must be carried out to fully characterize the mechanistic role of these factors in facial nerve outcome, this demonstrates the utility of applying novel data science techniques to uncover non-linear interactions between variables which may have real-world, clinical relevance.

### Tumor dimensions

As previously noted, tumor measurements utilized in our study were selected due to their relationships to surgical corridors, as well as having been shown to correlate well with tumor size by volumetric analysis in previous literature^10^. We found high ICC for all measurements, which was comparable to other reports in the literature on similar VS measurement tasks^26,27^. Although historically, an overall larger tumor size has been demonstrated to portend worse facial nerve function after microsurgical resection^19,20,28–30^, results of the present study identified the tumor dimension representing growth within the cerebellopontine angle between the mid-axis of the tumor and the brainstem as most predictive of facial nerve outcome. Our findings are consistent with prior literature, while providing further insight into possible mechanisms by which tumor size may influence facial nerve injury. A relatively larger tumor dimension within the cerebellopontine angle, between the brainstem and porus acusticus is postulated to result in more thinning and splaying of the facial nerve. This causes direct mechanical injury and makes the facial nerve more difficult to distinguish from tumor capsule and surrounding adherent arachnoid, placing the facial nerve at greater risk of iatrogenic injury^31^. Thus, our study builds on prior literature reporting greater tumor size as a predictor of facial nerve injury following vestibular schwannoma microsurgery, by suggesting that the tumor dimension representing growth within the cerebellopontine angle from the mid-axis of the tumor towards the brainstem has the greatest implication on facial nerve outcome. We did not identify any difference between our facial nerve preservation and facial nerve dysfunction groups when comparing this dimension. It is worth noting that we observed a relatively higher rate of Koos grade III and IV tumors compared to other published series, suggesting that this series may be skewed towards larger tumors overall. This may partially explain our inability to decipher a difference between facial nerve preservation and facial nerve injury groups based on tumor size. We anticipate that future studies including larger cohorts of patients might capture a relationship between facial nerve susceptibility to injury as this tumor dimension increases.

### Age

Older patient age has been previously shown to be predictive of facial nerve dysfunction, similar to our own findings^20,29^, though this remains controversial. While some studies have found no significant relationship between post-operative facial nerve function and age^32^, our study and others have identified a trend towards increasing age influencing unfavorable facial nerve outcomes following vestibular schwannoma microsurgery^33^. Others reporting on this finding have hypothesized on the influence of frailty, burden of comorbidities, decreased neurologic reserve resulting in reduced facial nerve rehabilitation potential^33^, and the confounding influence of age itself on facial nerve grading given that skin laxity and thinning may contribute to worse grading and/or worsened manifestations of facial nerve paralysis in elderly patients^34^. We further hypothesize that the basis of this relationship might be less favorable tissue dissection planes in patients of advanced age, placing older patients at greater risk of iatrogenic facial nerve injury. Although further detailed analysis of the role of age in facial nerve outcome on patients undergoing vestibular schwannoma microsurgery is beyond the scope of the current study, further study would certainly be valuable to confirm and better characterize the nature of this relationship. Our study further demonstrated additional unique features predictive of facial nerve outcomes which have not been previously identified. Our hypotheses regarding the role of BMI and case length are discussed further below.

### BMI

Interestingly, our model identified BMI and operative case length as being highly predictive of facial nerve outcome at 6 months post-operatively. To the best of our knowledge, these associations have not been clearly delineated in previous studies. One study examined facial nerve injury in the context of post-operative complications and the need for readmission or re-operation, finding no significant association to BMI^35^. However, as the authors note, facial nerve injury often occurs without the requirement for reoperation and readmission, thus is likely underrepresented in their analysis. Another study evaluated the influence of BMI on mean HB score pre-operatively (1.1 non-obese vs. 1.0 obese, p = 0.16) and post-operatively (1.9 non-obese vs. 1.7 obese, p = 0.32) finding no difference between obese and non-obese groups^36^. However, the timing of facial nerve function assessment is not clearly specified in this study and when facial function is modelled as a categorical variable (rather than continuous, summarized with mean HB scores), obese patients were more likely than non-obese patients to have HB scores equal to or greater than III (9.2% non-obese vs. 17.7% obese). The observed association between BMI and facial nerve dysfunction in our study may be seen as hypothesis-generating, and should be explored in future studies. It is possible that difficult surgical ergonomics in high-BMI patients make tumor dissection off of the facial nerve more difficult, placing patients at higher risk of dysfunction^37–39^. For example, in higher BMI patients, relatively higher mass of the neck and shoulder may further narrow an already small operative working corridor, which in addition to requiring less ergonomic positioning for tumor access, limits the dissection vectors and angles, and reduces range of motion and visibility. The increased utilization of endoscopes^40^ and exoscopes^41^ in lateral skull base surgery may eventually mitigate some of these constraints.

### Case length

Operative duration is identified as a key factor associated with facial nerve outcome in microsurgical resection of vestibular schwannomas in the present study—to our knowledge, this is the first such description of this association, however, this is consistent with previous studies in which prolonged operative duration has been shown to be associated with a higher rate of complications^42^. Our observed association of increased operative length being associated with a higher likelihood of facial nerve dysfunction may be reflective in part of the known association between tumor size and facial nerve outcomes, as a result of larger tumors having longer average operative durations. However, given that larger overall tumor size and individual tumor measurements in three dimensions (parallel to the posterior petrous bone, between central axis of tumor and porus acusticus, and from porus acusticus to distalmost extent of tumor growth within the IAC) were not found to be predictive of facial nerve dysfunction, other factors which may increase case length should be considered and investigated in future studies as the underlying mechanism of this association. Factors such as tumor hypervascularity^43^, adherence to the facial nerve perineurium, and the direction of facial nerve displacement may be reflected among difference in operative length across patients, and thus contribute to the observed differential risk of facial nerve dysfunction as it relates to case length^20^. These factors may serve as a surrogate for dissection complexity. Lastly, it is important to recognize that this algorithm, as any machine learning/artificial intelligence tool, is limited by the inputs. As such, there may be other confounding variables that influence facial nerve injury risk which were not captured in our data or analysis. Further study will be critical to better understand the myriad factors which may influence the role of case length on facial nerve outcome in vestibular schwannoma microsurgery.

A major strength of this study is the inclusion of patient cohorts from three hospitals across two health systems, increasing the generalizability of the resulting model. The model demonstrates an expected performance decay from 90.5 to 84% when assessed on unseen data from one of the included institutions. This level of performance decay both demonstrates the low likelihood of overfitting of this model and the relative reliability of the model in the real world (clinical) context. While the current model demonstrates good accuracy while avoiding overfitting, we recognize that performance will continue to improve in the deployment phase as further data is collected at external sites and through future prospective validation with patient data from the participating institutions (Supplementary Fig. 2). While we appreciate the tremendous benefit of multi-center data collection to enhance reproducibility, generalizability and clinical translation of our algorithm, we also recognize that as we increase the number of participating centers and expand to include institutions outside of our region, hospital-related factors (setting, level of care, equipment, etc.) and surgeon-related factors (patient selection, preferred surgical approach, years of experience, etc.), will need to be considered and evaluated in this stage of deployment^44^.

A limitation of the present study is an overall small proportion of patients with facial nerve dysfunction, which likely limited the statistical significance of associations which may have clinical relevance, as well as our ability to further stratify patients into different grades of facial function (i.e. HB I–VI). As vestibular schwannoma is a relatively rare disease entity, expanding our database with each currently participating institution will occur at a rate of roughly 30–60 patients per year, thus increasing the time to build a dataset robust enough to meaningfully improve the model metrics and generalizability. However, we aim to overcome this limitation through dissemination of our results and the current iteration of the algorithm—we aim to expand this work to include additional intuitions both nationally and internationally with the goals of improving statistical power, and further increasing the generalizability of this work. As additional validation is performed, we anticipate that the machine learning lifecycle will re-start, including further iterations of model evaluation and tuning to further improve performance.

As previously noted, the current iteration of this algorithm was developed based on manual tumor measurements that have been shown to have strong reproducibility and correlation with volumetric analysis throughout the vestibular schwannoma literature. However, accelerated deployment could be expedited through automated tumor segmentation—several such promising tools have recently been developed for vestibular schwannoma, however, in all cases the authors acknowledge that these will require further validation before implementation^45–48^. This approach has shown significant promise in other medical contexts, particularly in developing strategies for automating chest X-ray review during the COVID-19 pandemic^49,50^, and in the identification of concerning vs. benign gastrointestinal polyps^51,52^. Lastly, as data science techniques are increasingly applied in medicine, no discussion of their implementation in this context is complete without considering the protection of patient privacy and confidentiality. The algorithm we present here is run locally and completely offline. However, cloud-based automation offers several advantages that must be weighed against the potential for data leakage—strategies for obviating security concerns while maintaining the flexibility, reliability, and accelerated deployment afforded by these tools are under development. A full discussion of such methods is beyond the scope of this paper, but can be further explored in recent works by Mei et al.^53^ and Wu et al.^54^, among others.

It is our goal that this algorithm will ultimately be utilized as a clinically valuable tool for stratifying an individual patient’s risk of facial nerve injury, aiding in pre-operative counseling about treatment approach (watchful waiting vs. radiosurgery vs. microsurgical resection) and timing. Importantly, the model was evaluated via accuracy, sensitivity and specificity given the common utilization of these as metrics of test performance in the clinical setting. In this specific context, we interpret the 90% accuracy to be excellent compared to the 85% accuracy which has been referenced as a benchmark of acceptable performance^15^—we further anticipate improved accuracy and generalizability performance (less performance decay), with the addition of validation examples during deployment. In addition, the sensitivity and specificity of 90% and 90% represent that the model performs equally well at predicting which patients are likely to have complete facial nerve preservation as it does at predicting which patients are likely to have facial nerve dysfunction. We anticipate that further validation through collaboration with additional centers which treat high volumes of vestibular schwannomas will continue to improve the model’s performance.

Recognizing that clinicians and patients with little to no computer programming background may find it cumbersome to implement the algorithm, we plan to develop a graphical user interface to facilitate ease of use in both exploratory and clinical settings. This concept has been applied in other areas of medicine to facilitate a user-friendly implementation of artificial intelligence in the clinical environment^55,56^.

## Future directions

Traditionally, tumor size has been the single most important factor in counseling patients regarding their risk of facial nerve injury. Importantly, our findings suggest that additional patient-, tumor- and surgery-related factors might influence the likelihood of facial nerve injury in vestibular schwannoma microsurgery. The finding that the tumor dimension representing the mid-axis of the tumor to the brainstem is important in deciphering which patients are likely to experience facial nerve injury builds on existing literature which has found tumor size to be a critical determinant of facial nerve outcome by offering more granularity to the description of the potential role of tumor size. In addition, our finding that increased age is of relative importance in predicting facial nerve outcome adds to existing literature finding the same. Lastly, the findings that elevated BMI and longer case length are of relative importance in predicting the likelihood of facial nerve dysfunction following vestibular schwannoma microsurgery are novel to this study and hypothesis-generating. For all of the described factors, future validation in independent cohorts are worthwhile endeavors. In addition, further exploration of variables not represented in this study, but which might influence facial nerve outcome in vestibular schwannoma microsurgery may continue to build on the findings presented here towards improving patient outcomes.

## Conclusions

Here, we have described the development of a multi-institutionally derived machine learning algorithm to predict the likelihood of facial nerve injury following microsurgical resection of VS. Our model demonstrated a high degree of accuracy, and was able to identify novel predictors of facial nerve dysfunction following microsurgical resection of VS. The model will be further developed as a clinical tool for predictions of facial nerve outcome. With the inclusion of additional national and international institutions to improve generalizability, our ultimate goal is to utilize this tool for counseling patients about surgical risk, and aid in surgical decision-making. More broadly, while further evaluation is necessary to fully understand the mechanistic implications of the features identified, this analysis has demonstrated the utility of machine learning in identifying clinically relevant factors which may otherwise evade elucidation via linear statistical methods, such as comparisons of measures of centrality.

### Supplementary Information

Supplementary Information.

## Data Availability

In accordance with the University of Pennsylvania Institutional Review Board requirements for study exemption, the research data supporting this project may not be publicly shared. De-identified data may be shared upon reasonable request following execution of a Data Usage Agreement. Please reach out to corresponding author, SMHA, with any inquiries.
