# Supplementary material for: A multi-institutional machine learning algorithm for prognosticating facial nerve injury following microsurgical resection of vestibular schwannoma
Source: Sci Rep. 2024 Jun 5;14:12963. doi: 10.1038/s41598-024-63161-1 (PMC11153496; doi:10.1038/s41598-024-63161-1)
Supplement: Supplementary file 1 — Supplementary Information. [file 41598_2024_63161_MOESM1_ESM.pdf]

Supplemental Information for "A Multi-institutional Machine Learning Algorithm for Prognosticating Facial Nerve Injury Following Microsurgical Resection of Vestibular Schwannoma"

Authors: Sabrina M. Heman-Ackah MD DPhil (Oxon) MSE[1,2], Rachel Blue MD[1], Alexandra E. Quimby MD MPH[3,4], Hussein Abdallah[5], Elizabeth M. Sweeney PhD[6], Daksh Chauhan[7], Tiffany Hwa MD[3], Jason Brant MD[3,8], Michael J. Ruckenstein MD MSc FACS[3], Douglas C. Bigelow MD[3], Christina Jackson MD[1], Georgios Zenonos MD[9], Paul Gardner MD[9], Selena E. Briggs MD PhD MBA FACS[10,11], Yale Cohen PhD[2,3,7], John Y.K. Lee MD[1,3]

1. University of Pennsylvania, Department of Neurosurgery, Philadelphia, PA
2. University of Pennsylvania, Department of Bioengineering, Philadelphia, PA
3. University of Pennsylvania, Department of Otorhinolaryngology, Philadelphia PA
4. SUNY Upstate Medical University Hospital, Department of Otolaryngology and Communication Sciences, Syracuse, NY
5. University of Pittsburgh, School of Medicine, Pittsburgh, PA
6. University of Pennsylvania, Department of Biostatistics, Epidemiology & Informatics, Philadelphia, PA
7. University of Pennsylvania, Perelman School of Medicine, Philadelphia, PA
8. Corporal Michael J. Crescenz VAMC, Philadelphia, PA
9. University of Pittsburgh, Center for Cranial Base Surgery, Pittsburgh, PA
10. MedStar Washington Hospital Center, Department of Otolaryngology, Washington, DC
11. Georgetown University, Department of Otolaryngology, Washington, DC

Corresponding Author:

Sabrina M. Heman-Ackah MD DPhil (Oxon) MSE
University of Pennsylvania, Department of Neurosurgery
3400 Civic Center Boulevard
Perelman Center for Advanced Medicine, 15th Floor
Philadelphia, PA 19104
E-mail: sabrina.heman-ackah@pennmedicine.upenn.edu
Phone: 919-338-9437

Contents:

- Supplemental Figure 1: Method for Tumor Measurements
- Supplemental Figure 2: The Machine Learning Lifecycle
- Supplemental Figure 3: Data Visualization
- Supplemental Table 1: Description of Tumor Measurements

A

B

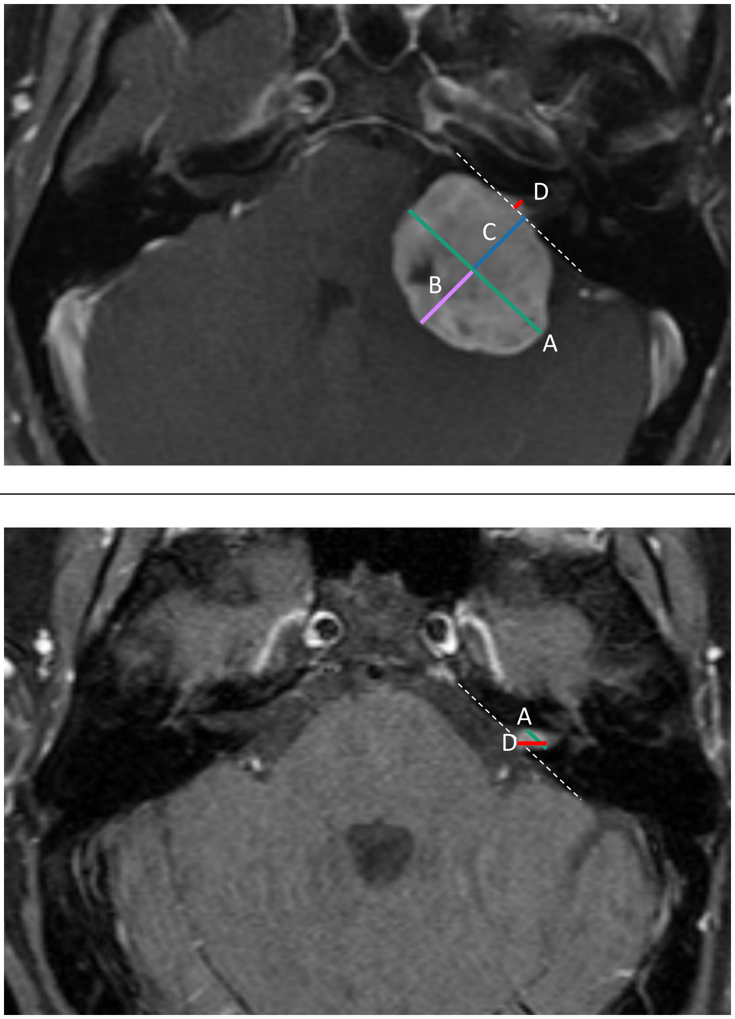

Supplemental Figure 1. Examples of tumor dimension measurements made in tumors with extension in the CPA (A) and those which were entirely intracanalicular (B). Measurement A is shown in teal, B in purple, C in blue and D in red. Note that for entirely intracanalicular tumors, B and C measured 0.

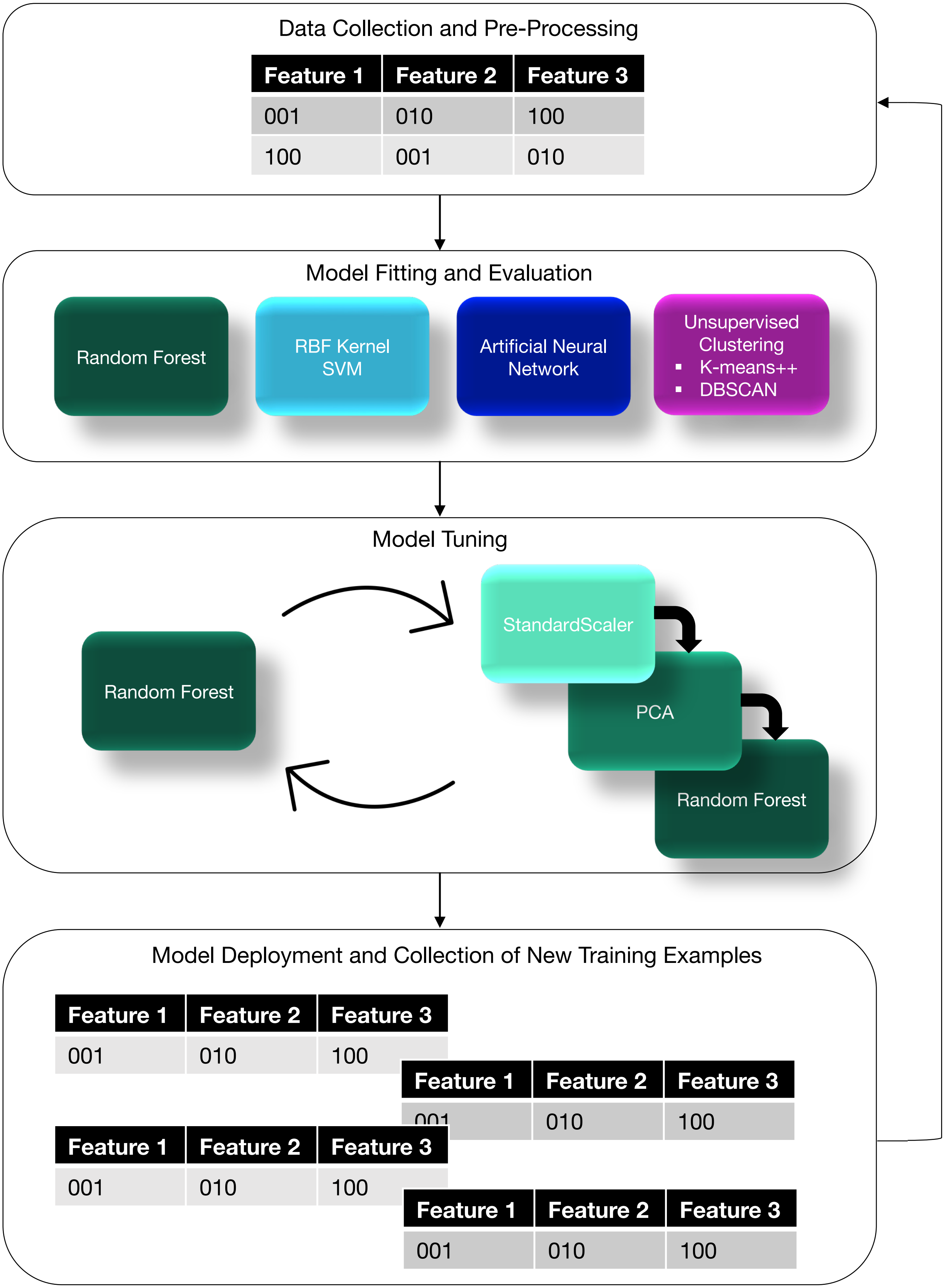

Supplemental Figure 2. The Machine Learning Lifecycle

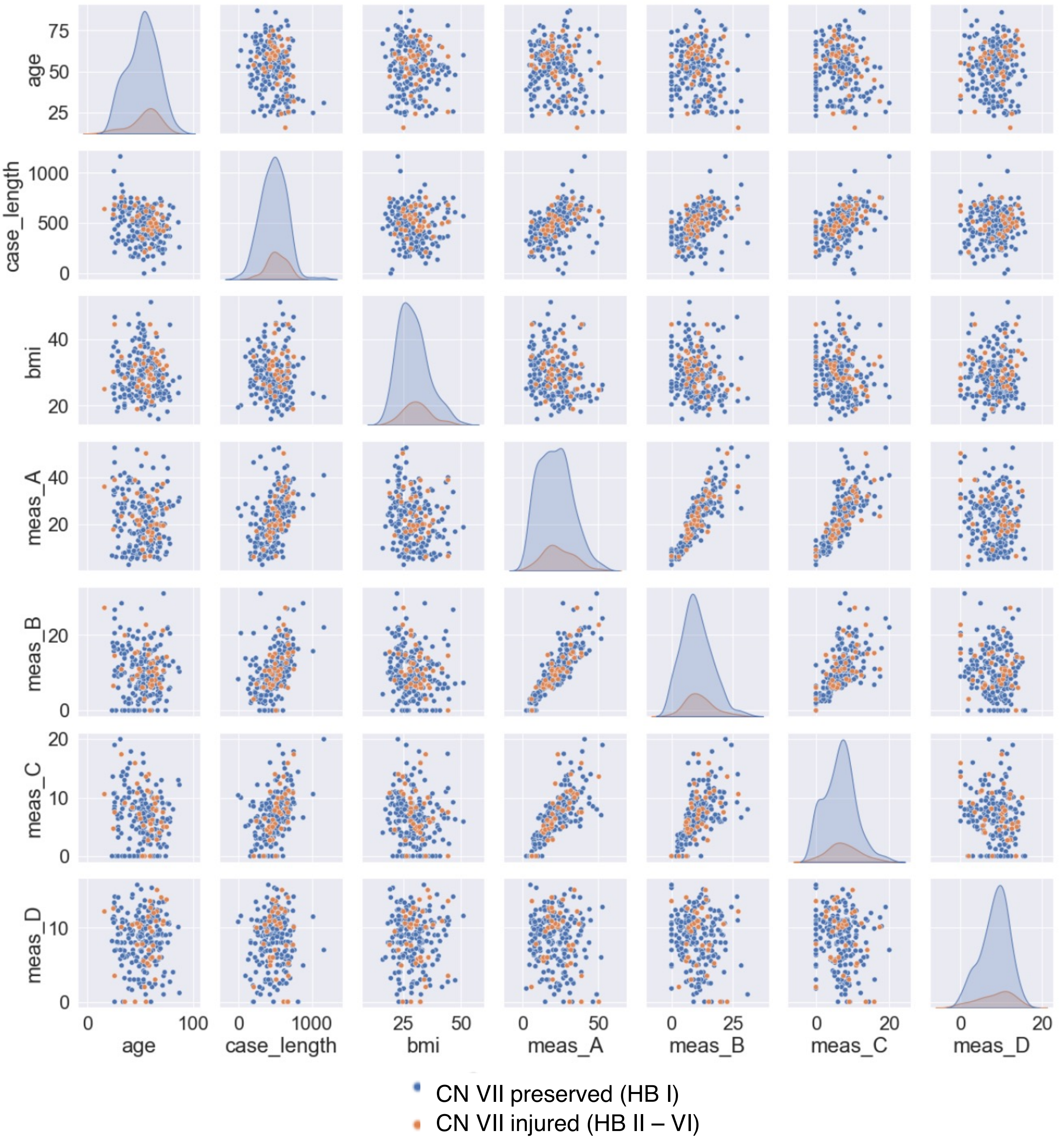

Supplemental Figure 3. Data visualization. To determine if patients with facial nerve preservation and those with facial nerve damage classes were linearly separable across any feature dimension, the data was visualized along all continuous variable feature axes using a pairplot. The cranial nerve VII damage class is shown in orange, while the cranial nerve VII preservation class is shown in blue. The classes are not clearly linearly separable, and the preponderance of cases in which cranial nerve VII is preserved results in a class imbalance which was addressed via synthetic minority oversampling technique (SMOTE) as described in the methods section.

Supplemental Table 1. Description of Imaging Measurements		
**Measurement**	**Description**	**ICC**
**A**	Greatest tumor dimension parallel to the petrous bone.	99.5945
**B**	Greatest perpendicular tumor dimension measuring from the medial-most (CPA) aspect of tumor to the line generated in measurement A	98.0340
**C**	Perpendicular tumor dimension spanning the line generated in measurement A to the porus acusticus	96.2142
**D**	Greatest perpendicular tumor dimension spanning from the porus acusticus to the lateralmost extent of contrast enhancement in the IAC.	90.5942
